# Attention-Based Fully Gated CNN-BGRU for Russian Handwritten Text

**DOI:** 10.3390/jimaging6120141

**Published:** 2020-12-18

**Authors:** Abdelrahman Abdallah, Mohamed Hamada, Daniyar Nurseitov

**Affiliations:** 1 Department of Machine Learning & Data Science, Satbayev University, 050013 Almaty, Kazakhstan; 2 National Open Research Laboratory for Information and Space Technologies, Satbayev University, 050013 Almaty, Kazakhstan; nurseitovdb@gmail.com; 3 Department of Information System, International IT University, 050000 Almaty, Kazakhstan; Dr.mmhamada@gmail.com

**Keywords:** handwriting recognition, fully gated convolutional neural networks, bidirectional gated recurrent unit, deep learning

## Abstract

This article considers the task of handwritten text recognition using attention-based encoder–decoder networks trained in the Kazakh and Russian languages. We have developed a novel deep neural network model based on a fully gated CNN, supported by multiple bidirectional gated recurrent unit (BGRU) and attention mechanisms to manipulate sophisticated features that achieve 0.045 Character Error Rate (CER), 0.192 Word Error Rate (WER), and 0.253 Sequence Error Rate (SER) for the first test dataset and 0.064 CER, 0.24 WER and 0.361 SER for the second test dataset. Our proposed model is the first work to handle handwriting recognition models in Kazakh and Russian languages. Our results confirm the importance of our proposed Attention-Gated-CNN-BGRU approach for training handwriting text recognition and indicate that it can lead to statistically significant improvements (*p*-value < 0.05) in the sensitivity (recall) over the tests dataset. The proposed method’s performance was evaluated using handwritten text databases of three languages: English, Russian, and Kazakh. It demonstrates better results on the Handwritten Kazakh and Russian (HKR) dataset than the other well-known models.

## 1. Introduction

Today, handwriting recognition is a crucial task. Providing solutions to this problem will facilitate business process automation for many companies. A clear example is a postal company, where the task of sorting a large volume of parcels is a complicated issue.

Handwriting recognition (HWR) or Handwritten Text Recognition (HTR) is a machine’s capacity to obtain and interpret intelligible handwriting information from such sources as paper documents, images, touchscreens, and other tools. Offline HTR is the task of converting letters or words into images and then into a digital text. The input is a variable two-dimensional image, and the output is a sequence of characters. It provides excellent human-machine contact, and it can support the automated processing of handwritten documents. It also considers a sub-task of Optical Character Recognition (OCR), mainly focusing on extracting text from scanned documents and natural scene images. The recognition of Russian handwriting poses specific challenges and advantages and has been more recently addressed than recognizing texts in other languages.

First, the main differences here are that the set of words for recognition is limited to words that can occur in addresses; secondly, handwritten texts are written on a monotonous background, which significantly facilitates the segmentation process. However, other aspects of text recognition are general and can be considered regardless of their specific application. For example, an image of an envelope, like an image of any other handwritten document, cannot be directly used for address recognition since the system must first determine where the text is located; separate it from the background; segment text by words; and normalize words so that they are free of spatial transformation. Only after these procedures can the data be used to build descriptors, which are input data for the recognition model.

The main developments in the field of HTR for postal correspondence were studied. They are mainly aimed at solving the problems of determining the area of interest, text segmentation, removal of background noises that interfere with the work with the text, such as lost or unclear fragments, spots on paper, skew detection, as well as training artificial intelligence to recognize written text in the used language. The most frequently used recognition models in this context are analyzed, namely models based on HMM, hybrid Markov models (Hybrid HMM), convolutional (CNN), and recurrent neural networks (RNN).

Previous approaches to the offline HTR use Hidden Markov Models (HMM) for transcription tasks [1], extracting features from images using a sliding window and then predicting character labels with an HMM [2], which is the prevalent automatic speech recognition approach [3]. HMM’s key benefits are their probabilistic nature, suitability for noise-corrupted signals like speech or handwriting, their computational foundations using efficient algorithms to change the model parameters automatically and iteratively. The success of HMM led many researchers to expand it to handwriting recognition, describing each word picture as a series of remarks. Two approaches can be differentiated according to how this representation is performed: implicit segmentation [4,5], which leads the system to search for the image with components that match classes in its alphabet, also termed as recognition-based segmentation, and explicit segmentation, which involves a segmentation algorithm to divide words into simple units such as letters [6,7]. Despite the mentioned benefits of the HMM approach, there are some limitations [8,9] compared to the new models that are using an encoding-decoder network, which combines a convolutional neural network (CNN) with a bidirectional recurrent neural network and with a Connectionist Temporal Classification (CTC) output layer [10,11]. Inspired by the latest advances in machine translation [12,13], automated question answer [14], image captioning [15], sentimental text processing [16], and speech recognition [17], we believe that encoder–decoder models with attention mechanisms [18,19] will become the new state-of-the-art for HTR tasks.

Attention-based methods have been used to help the networks learn the correct features, focus on the right features, and determine an alignment between an image pixel and target characters [20]. Attention increases the network’s capacity to collect the most critical information for every part of the output sequence. Furthermore, attention networks can model language structures in the output sequence instead of mapping the input to the correct output [17]. We proposed extensions to attention-based recurrent networks that allow them applicable to handwritten recognition. Handwritten recognition can be considered to be learning to generate a sequence. In our research, attention-based models are tested on variety of datasets, such as (HKR, IAM, etc.). The attention mechanism assigns or weighs the sequences provided by the trained extraction mechanism of the function for all possible features in the input sequence, then the weighted vector function helps to generate the next output sequence.

In this research, our contribution is to present a novel attention-based fully gated convolutional recurrent neural network, tested in Kazakh and Russian dataset [21], using the following data:

- Handwritten samples (forms) of keywords in Kazakh and Russian (Areas, Cities, Village, etc.).
- Handwritten Kazakh and Russian Cyrillic alphabet.
- Handwritten samples (Forms) of poems in Russian.

The main results of our work are summarized as follows: (i) For the first time, the use of gated convolutional neural network for the HTR task is studied; (ii) The effective attention mechanism and bidirectional gated recurrent neural network for the HTR task is developed; (iii) The problem of achieving results compatible with other models by keeping the number of parameters small for Attention-Gated-CNN-BGRU is considered; (iv) To verify the efficiency of our method, comprehensive ablation and comparative studies are carried out. Our proposed HTR system achieves state-of-the-art performance on the public HKR dataset [21].

The related work on Offline Handwriting Text Recognition is considered in Section 2. Section 3 demonstrates the attention-based, fully gated, convolutional recurrent neural network. Section 4 and Section 5 present the experimental results and analysis of the tested data obtained from Kazakh and Russian dataset; conclusions and remarks are given in Section 6.

## 2. Related Work

Handwritten text recognition approaches can be divided into specific categories: HMM-based approaches and RNN-based approaches. We discuss the strategies for each of these significant classes.

In offline HTR, the input features are extracted and selected from images, then artificial neural network (ANN) or HMM are used to predict the probabilities and decode them for the final text. The main disadvantage of HMMs is that they cannot predict long sequences of data. HMMs have been commonly used for offline HTR because they have achieved good results in automatic speech recognition [22]. The basic idea is that handwriting can be perceived as a series of ink signals from left to the right, similar to the acoustic signals invoice sequence. The inspiration for the hybrid HMM research models came from:

1. offline HTR [23],
2. offline HTR using conventional HMMs [24],
3. automatic speech recognition using hybrid HMM/ANN models [25],
4. and online HTR [26].

On the other hand, RNNs such as gated recurrent unit (GRU) [27] and long short-term memory (LSTM) [28] can solve this problem. RNN models have shown remarkable abilities in sequence-to-sequence learning tasks such as speech recognition [29], machine translation [30], video summarizing [31], automated question answer [14], and others.

To transform a two-dimensional image for offline HTR, it is necessary to take the image as a vector and forward it to an encoder and decoder. The task is solved by HTR, GRU, and LSTM, which take information and feature from many directions. These handwriting sequences are fed into RNN networks. Due to the use of Connectionist Temporal Classification (CTC) [32] models, the input feature requires no segmentation. One of the CTC algorithm’s key benefits is that it does not need any segmented labeled data. The CTC algorithm allows us to use data alignment with the output.

Anshul Gupta et al. [33] performed an analysis of various feature-based classification strategies to recognize offline handwritten characters. It was proposed to use an Optical Character Recognition technique after experimentation. The approach proposed involves the segmentation of a handwritten word using heuristics and artificial intelligence. Three Fourier descriptor combinations are used in parallel as vectors of the features. A support vector machine (SVM) is used as a classifier. Using the lexicon to verify the validity of the predicted word, post-processing is performed. It is found that the results obtained by using the proposed CR system are satisfying. It also tried a support vector machine (SVM) as a classifier on the same feature set. It achieved 98% classification accuracy on the training data set and 62.93% on the test data set.

Bianne Bernard et al. [34] created an effective system of word recognition resulting from the combination of three handwriting recognizers. A hybrid framework’s key component is an HMM-based recognizer that considers complex and contextual knowledge for better writing device modeling. A state-tying method based on decision tree clustering was implemented for modeling of the contextual units. Decision trees are built according to a collection of expert questions about how characters are created. Performance of the Proposed Systems on the IAM Test Database for HHM-based context-independent is 64.6% recognition rate, HHM-based context-dependent is 67.3%, and HHM-based proposed combination is 78.1% recognition rate.

Theodore Bluche et al. [18] presented an attentive model for the identification of end-to-end handwriting. The model was inspired by the recently introduced differential models that focus on voice recognition, image captioning, or translation. The key difference with a multidimensional LSTM network is the implementation of hidden and overt focus. Their main contribution to identifying handwriting is illustrated in automated transcription without prior line segmentation, which was imperative in the previous approaches. The machine can also learn the order of reading, and it can handle bidirectional scripts such as Arabic. They performed tests on the popular IAM database and announced promising results to full paragraph transcription. The proposed work achieved for one word 12.6% Character Error Rate(CER), for two words, 9.4% CER, for three words, 8.2% CER and for four words, 7.8% CER.

Theodore Bluche et al. [35] proposed new neural network architecture for state-of-the-art handwriting recognition as an alternative to recurrent neural networks in multidimensional long-term memory (MD-LSTM). The CNN model and a bidirectional LSTM decoder were used to predict sequences of characters. The research aims to generate generic, multilingual, and reusable features with the convolutional encoder, leveraging more data for learning transfer. The architecture is also motivated by the need for fast GPU training and quick decoding on CPUs. The results are competitive with the previous version of A2iA Text-Reader [36], and give 15.4%, 17.2%, 17.6% and 19.5% for accurate, fast, fast-small AND faster-small models, respectively.

Joan Puigcerver et al. [37] fulfilled state-of-the-art offline handwritten text recognition, mainly relying on long-term multidimensional memory networks. The long-term, two-dimensional dependencies, theoretically represented by multidimensional recurrent layers, may not be necessary to achieve a good accuracy of recognition, at least in the lower layers of architecture. In this study, an alternative model is considered, relying only on convolutional and one-dimensional recurrent layers that achieve better or comparable results than those of the current state-of-the-art architecture and run much faster. Furthermore, it was found that random distortions as synthetic data significantly improve the model precision. 1D-LSTM model achieves 5.1% CER on validation and 5.7% CER on the test. 2D-LSTM achieves 8.2% CER on validation and 8.3% CER on the test.

Zi-Rui Wang et al. [38] proposed a novel WCNN-PHMM architecture for offline handwritten Chinese text recognition. It is proposed to address two key issues: the extensive vocabulary of Chinese characters and the diversity of writing styles. By combining parsimonious HMM based on the state of unsupervised learning based on the writer’s code, the approach demonstrates its superiority to other state-of-the-art methods based on both experimental results and analysis. It achieves a relative 16.6% CER over the conventional CNN-HMM without considering language modeling.

Nam Tuan Ly et al. [39] presents a model of attention-based convolutional sequence to sequence (ACseq2seq) for recognizing an input image of multiple text lines without explicit line segmentation from Japanese historical documents. There are three main parts to the recognition system: a feature extractor using convolutional neural network (CNN) to extract a sequence of features from an input image; an encoder that uses bidirectional Long Short-Term Memory (BLSTM) to encode the sequence of features; and a decoder that uses a unidirectional LSTM with the mechanism of attention to generate the final target text based on the relevant features involved. In the test set for level 2 and level 3, the proposed ACseq2seq model achieved a 4.42% and 12.98% character error rate, respectively.

Lei Kang et al. [40] implemented Convolve, Attend and Spell in this paper, an attention-based seq2seq model for handwritten word recognition. There are three key components of the proposed architecture: an encoder consisting of a CNN architecture such as the VGG-19-BN and a bidirectional GRU, an attention mechanism devoted to the related characteristics, and a decoder generated by a single-directional GRU capable of spelling the corresponding word, character by character. The proposed model achieve 6.88% character error rate and 17.45% word error rate on IAM word-level dataset.

Arindam Chowdhury et al. [41] presented a novel approach that combines a deep convolutional network with a recurrent encoder–decoder network to map an image to a sequence of characters corresponding to the text present in the image. Using Focal Loss, the entire model is trained end-to-end, an improvement over the traditional Cross-Entropy loss that solves the issue of class imbalance inherent to text recognition. The Beam Search algorithm is used to boost the decoding ability of the model, which searches for the best sequence from a set of hypotheses based on a common distribution of individual characters. The proposed model achieves 8.1% character error rate and 16.7% word error rate on IAM word-level dataset and on RIMES dataset achieve 3.5% character error rate and 9.6% word error rate

Zelun Wang et al. [42] research proposed a deep neural network model with an encoder–decoder architecture that converts math formula images into their LaTeX markup sequences. The encoder is a neural convolutional network that converts images into a group of maps of features. Before being unfolded into a vector, the function maps are supplemented with 2D positional encoding to better capture the spatial relationships of math symbols. The decoder is a stacked long-term bidirectional memory model integrated with the mechanism of soft attention that acts as a language model to convert the output of the encoder into a sequence of LaTeX tokens.

This is the first paper that uses the HKR dataset [21]. The HKR dataset was also proposed by the authors, which is the first publicly available Russian and Kazakh handwritten dataset. Until now, there has been no dataset in these languages available to researchers.

## 3. Proposed Model

Attention-Gated-CNN-BGRU focuses on the Cyrillic symbol extracted in handwritten form. An input is a cropped word image of a 1D sequence of characters or symbols. We propose a model based on the Attention-Gated-CNN-BGRU architecture with the number of parameters around (885,337); it has a high recognition rate, is more compact and faster, and has a lower error rate compared with the other models. The algorithm consists of six stages, which will be described as follows:

1. Preprocessing such as Resize with padding (1024 × 128), Illumination Compensation, and Deslant Cursive Images is performed. We then convert the raw data into a hierarchical data format (HDF5) files; this conversation helps us provide fast loading of the data.
2. Extract characteristics by using the CNN layers.
3. Bahdanau attention mechanism that makes the model pay attention to the inputs and relates them to the output.
4. The map features sequence by BGRU.
5. Calculate the loss function Connectionist Temporal Classification (CTC) [32].
6. Decode the output into the text format and perform post-processing to improve the final text.

### 3.1. Image Preprocessing

To minimize lighting irregularities in the image, such as light peaks and excessive shadows, the first approach, brightness and contrast correction, is applied. Other techniques such as lighting compensation are also useful in this case, as background and foreground noises are both eliminated by lighting. Contrast adjustment remaps image intensity values to the full display range of the data type. An image with good contrast has sharp differences between black and white.

The second approach, slant correction or de-slant, is meant to normalize the characters’ vertical tendency. The process identifies the slant angle of the writing on the vertical axis, then applies a transformation of the geometric image to change the observed angle. In HTR applications, slant correction is an important part of the normalization task. The slant is the deviation from the vertical direction of the vertical strokes of words. It is possible to extend the de-slanting process to many applications. It may assist the segmentation and identification of digits in natural scenes, license plates, zip codes, and handwritten texts of italic machine-printed characters.

The last approach, standardization (or normalization of the Z-score), aims to rescale the image characteristics (pixels) to result in an image with mean and variance values equal to 0 and 1, respectively.

### 3.2. Model

**Figure 1:**
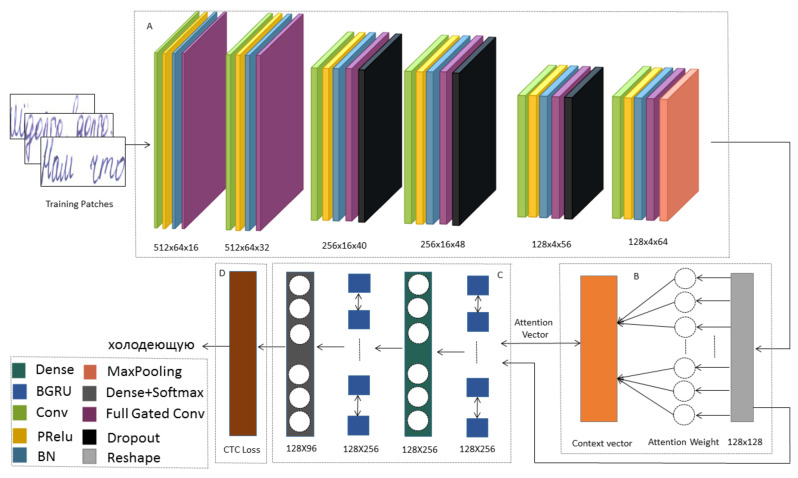
Attention-Gated-CNN-BGRU architecture for handwriting recognition. The system contains four main parts: (**A**) encoder, (**B**) attention block, (**C**) decoder, (**D**) CTC. An input image is converted into a series of constant feature vectors by the encoder. The attention block is a mechanism that provides a richer encoding of the source sequence that facilitates the building of a context vector, such that the decoder can use it. The decoder is processing feature sequences to predict sequences of characters. CTC of the output layer for RNN is used for sequence labeling problems.

The model is fed by the image through the Gated CNN, processed using the Bahdanau’s attention, with bidirectional GRU. Finally, GRU’s output matrix is passed to the Connectionist Temporal Classification (CTC [32]) to calculate the loss value and decode the output matrix into the final text. The model architecture, which has four primary parts: an encoder, an attention block, a decoder, and CTC, is shown in Figure 1.

#### 3.2.1. Encoder

##### Convolutional Blocks

The encoder receives the input and generates the feature vectors. These feature vectors hold the information and the characteristics that represent the input. The encoder network consists of 6 convolutional blocks that correspond to training to extract relevant features from the images. Each block consists of a convolution operation, which applies a filter kernel of size (3,3) in the first, second, fourth, and sixth blocks and (2,4) in the third and fifth block. Parametric Rectified Linear Unit (ReLU) and Batch Normalization are applied. To reduce overfitting, we also use Dropout at some of the convolutional layers [43] (with dropout probability equal to 0.2).

##### Gated Convolutional Layer

The idea of gate controls is to propagate a feature vector to the next layer. The gate layer looks at the vector feature’s value at the given position and the adjacent values and determines if it should be held or discarded at that position. It allows generic features to be computed across the entire image and filtered when the features are appropriate, depending on the context. The gate (*g*) layer is implemented as a convolutional layer with the Tanh activation layer. It is added to the input function maps (*x*). The output of the gate mechanism is the pointwise multiplication of the inputs and outputs of the gate.

(1) y=g(x)·x

**Figure 2:**
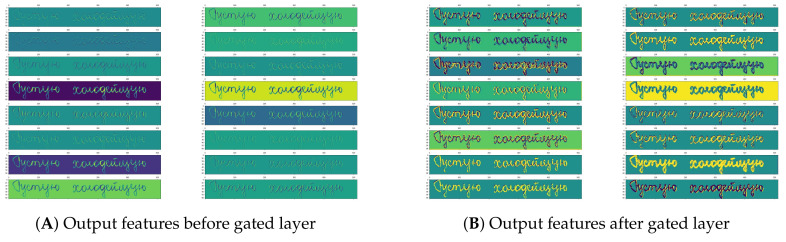
Visualization of the feature of a convolutional layer and gate layer. The gate layer looks at the vector feature’s value at the given position and the adjacent values and determines if it should be held or discarded at that position.

Figure 2 shows the feature maps of a real example of the feature before and after the gated layer. This example shows that the gated layer allows the feature to be more effective and excitatory.

#### 3.2.2. Decoder

The decoder is a bidirectional Gated Recurrent Unit (GRU) [20] RNN that processes feature sequences to predict sequences of characters. The feature vector contains 256 features per time-step, and the recurrent neural network propagates the information through the sequence. The GRU implementation of RNNs is employed as a gating mechanism in recurrent neural networks (RNN), almost like an extended LSTM unit without an output gate. GRU tries to unravel the matter of the vanishing gradient [44].

GRU can solve the vanishing gradients problem by using an update gate and a reset gate. The update gate can control the information that flows into the memory, and the reset gate can control the memory-flowing information. The gates for updating and resetting are two vectors that determine which information will be passed on to the output. Two vectors can be qualified to retain past knowledge or delete information unrelated to prediction. The GRU is similar to LSTM with a forgotten gate, but it contains fewer parameters because it lacks a gate for output. The output sequence of the RNN layer is a matrix of size 128 × 96.

#### 3.2.3. Attention Mechanism

The attention block is a mechanism that provides a richer encoding of the source *sequence* (*h*_1_,...,*h*_*s*_) that facilitates the building of a context vector (*c*_t_), such that the decoder can use it.

The attention block allows the model to pay attention to the most relevant information in the source. The source sequence’s hidden state is obtained from the encoder for each input time-step instead of the hidden state for the final time-step. The attention weights ($\alpha_{ts}$) calculated based on the encoder hidden state ${\bar{h}}_{s}$, and the sequence of decoder hidden state vectors $h_{t}$. The context vector (ct) is computed by the current attention weights ($\alpha_{ts}$) and the sequence of encoded hidden state vectors ${\bar{h}}_{\overset{'}{s}}$ in the following equation:

(2) $$\begin{array}{c} Attention\;weight\left(\alpha_{ts}\right)=\frac{exp(score\left(h_{t},{\bar{h}}_{s}\right)}{\sum_{\overset{'}{s}=1}^{S}exp\left(score\left(h_{t},{\bar{h}}_{\overset{'}{s}}\right)\right)} \end{array}$$

(3) $$\begin{array}{c} Context\;vector\left(c_{t}\right)=\sum_{s}\alpha_{ts}{\bar{h}}_{s} \end{array}$$

In the target sequence, a context vector(ct) is constructed explicitly for every output word. First, using a neural network, every hidden state from the encoder is graded and then normalized to have a probability over the encoders’ hidden states. Finally, the probabilities are used to calculate a weighted sum of the encoder’s hidden states to provide a context vector that should be used in the decoder. The attention layer produces outputs of dimension 128 × 256.

(4) $$\begin{array}{c} Attention\;vector\left(a_{t}\right)=f\left(c_{t},h_{t}\right)=tanh\left(W_{c}\left[c_{t};h_{t}\right]\right) \end{array}$$

(5) $$\begin{array}{c} score\left(h_{t},{\bar{h}}_{s}\right)=\upsilon_{a}^{⊤}tanh\left(W_{1}h_{t}+W_{1}{\bar{h}}_{s}\right) \end{array}$$

where both $\upsilon_{a}^{⊤}$ and $W_{c}$ are weight matrices to be learned in the alignment model, *S* is source sequence length, ${\bar{h}}_{\overset{'}{s}}$ is encoder hidden state and $h_{t}$ is decoder hidden state network.

#### 3.2.4. Connectionist Temporal Classification(CTC)

The Connectionist Temporal Classification (CTC) of the output layer for RNN is used for sequence labeling problems. There is no alignment between the inputs and the target labels. Neural networks need different training goals for each section of the input sequence or time-step.

CTC has two significant consequences. First, it ensures that the training data must be pre-segmented to set the goals. Secondly, as the network generates only local classifications, the global aspects of the sequence (such as the probability of two labels occurring consecutively) must be modeled externally. Indeed, the final label sequence cannot be inferred reliably without some post-processing. CTC is achieved by allowing the network to make label predictions at any time in the input sequence, provided that the overall label sequence is correct. CTC can eliminate the necessity of pre-segmented data because it is no longer essential to align the labels with the input. CTC also offers complete label sequence probabilities directly, ensuring that no additional post-processing is required for using the network as a time classifier.

While training the neural network, the CTC is given the RNN output matrix and the ground-truth text, and it computes the loss value. While inferring, the CTC is only given the matrix, and it decodes it into the final text. Both the ground-truth text and the recognized text length can be mostly 96 characters long.

## 4. Experiment Setup

### 4.1. Data

The handwritten Kazakh and Russian database [21] can serve as a basis for research on handwriting recognition. It contains Russian Words (Areas, Cities, Villages, Settlements, Streets) written by a hundred different writers. It also incorporates the most popular words in the Republic of Kazakhstan. A few preprocessing and segmentation procedures have been developed together with the database. They contain free different handwriting forms. The database is prepared to provide training and testing set for Kazakh and Russian word-recognition research. The database consists of more than 1400 filled forms. There are approximately 63,000 sentences, more than 715,699 symbols, and there are approximately 106,718 words.

**Figure 3:**
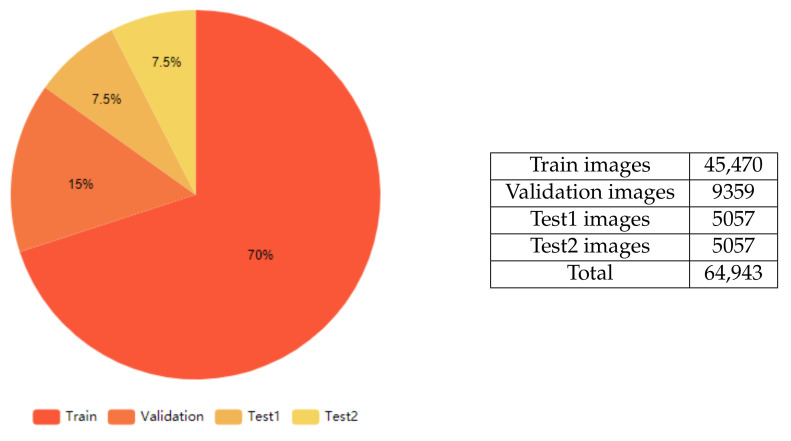
Training, validation and testing dataset. The dataset was split into three datasets as follows: Training (70%), Validation (15%), and Testing (15%). The test dataset was equally split into two sub-datasets (7.5% each): the first dataset was named Test1 and consisted of words that did not exist in Training and Validation datasets; the second was named Test2 and was made up of words that exist in Training dataset but with totally different handwriting styles.

Due to the scarcity of public data for Kazakh and Russian languages, the HKR dataset [21] is used in this work, which contains 64,943 text lines is divided, as shown in Figure 3.

**Figure 4:**
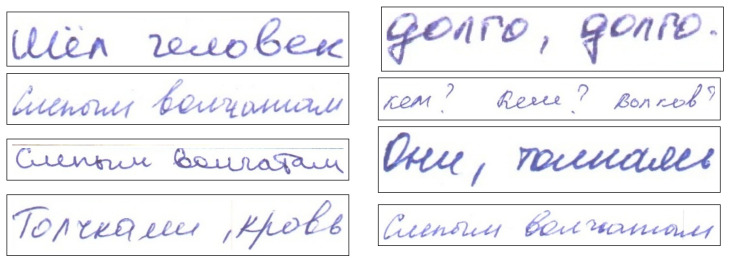
Some sample images from the HKR dataset.

The basis for this work’s dataset was made up of different words (or short sentences) written in Russian and Kazakh languages (approximately 95% of Russian and 5% of Kazakh words/sentences, respectively). These languages use Cyrillic and share the same 33 characters. Besides these characters, the Kazakh alphabet also contains nine additional specific characters. The dataset of distinct words/sentences was boosted by applying various handwriting styles ( approximately 50–100 different persons) to each of these other words. These procedures resulted in a final dataset with a large number of handwritten words/sentences. After that, the final dataset was split into three datasets as follows: Training (70%), Validation (15%), and Testing (15%). The test dataset was equally split into two sub-datasets (7.5% each): the first dataset was named TEST1 and consisted of words that did not exist in Training and Validation datasets; the second was named TEST2 and was made up of words that exist in Training dataset but with totally different handwriting styles. The primary purpose of splitting the Test dataset into TEST1 and TEST2 was to check the accuracy of the difference between recognizing unseen words and the other words, which were seen in the training phase but with unseen handwriting styles. After training, validation, and testing datasets were prepared, the models were trained, and a series of comparative evaluation experiments were conducted. Figure 4 shows examples of images in the dataset.

### 4.2. Training

Attention-Gated-CNN-BGRU are trained to minimize the CTC function’s validation loss value. The optimization with stochastic gradient descent is performed, using the RMSProp method [45] with a base learning rate of 0.001 and mini-batches of 32. Also, early stopping with patience 20 is applied, we wanted to monitor the validation loss at each epoch, and when the validation loss does not improve after 20 epochs, training is interrupted.

## 5. Result

### 5.1. Experiments

The proposed and tested models have all been implemented using the Tensorflow library [46] for Python, which allows transparent use of highly optimized mathematical operations on GPU through Python. A computational graph is defined in the Python script to define all necessary operations for the specific computations. The tensors are then evaluated, and Tensorflow runs the necessary part of the computational graph implemented C code on the CPU or the GPU if any is made available to the script. The operations have a supported GPU implementation. Although Tensorflow supports the use of multiple GPUs, in our project, we use only one GPU for each test run to make the processing easier. The experiments were run on a machine with 2× “Intel(R) Xeon(R) E-5-2680” CPUs and 4× “NVIDIA Tesla k20x”.

### 5.2. Comparison with State-of-the-Art on HKR Dataset

This section presents the results of applying the research model and compares its performance with the other published models, which used different datasets to achieve a state-of-the-art scientific comparison (Bluche, and Puigcerver) [35,37]. The dataset is divided into four parts: training, validation, Test1, and Test2. Attention-Gated-CNN-BGRU and other models are tested via double tests of datasets, as shown in Table 1. This Table shows that there is quite a difference between Attention-Gated-CNN-BGRU and the Puigcerver model in the character error rate (CER) because the Puigcerver model has many parameters (around 9.6 million) and overfitting after 30–50 epochs.

Our network was trained from scratch because there is no pre-training model or transfer from another dataset used before. The standard performance measures are used for all the results presented: the character error rate (CER) and word error rate (WER) [47]. The CER is determined as the distance from Levenshtein, which is the sum of the character substitution (*S*), insertion (*I*), and deletions (*D*) required to turn one string into another, divided by the total number of characters in the ground-truth word (*N*).

(6) $$\begin{array}{c} CER=\frac{S+I+D}{N} \end{array}$$

**Figure 5:**
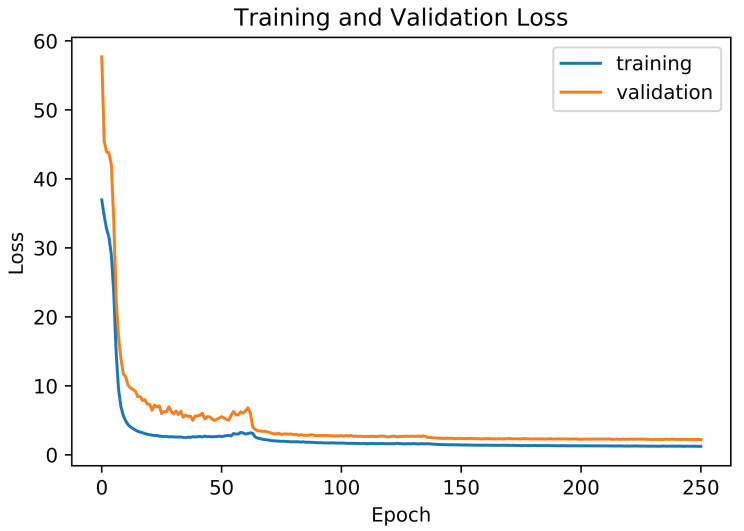
Training and validation loss.

Similarly, the WER is calculated as the sum of the number of the term substitutions ($S_{w}$), insertion ($I_{w}$), and deletions ($D_{w}$), which is necessary for the transformation of one string into another, and divided by the total number of ground-truth terms ($N_{w}$). Attention-Gated-CNN-BGRU was trained to minimize the validation loss value of the CTC function. Training and validation loss is shown in Figure 5.

Table 2 presents randomly selected samples obtained on the HKR dataset. An impressive result is shown in these examples. In the first example, only one character was not detected; it is the letter “щ”. In the second example also only one character, “Ь” was not detected, and the two letters are the same, but one is capital, and the other is small “б, Б”. The third example has many errors in characters because the style of writing is contacted, and some characters in Russian are the same if contacted, for example, “иц,щ”. The rest of the examples are correct. Attention-Gated-CNN-BGRU not only detects characters, but it can also detect symbols like (’.’,’!’,’?’, etc.).

(7) $$\begin{array}{c} WER=\frac{S_{w}+I_{w}+D_{w}}{N_{w}} \end{array}$$

Table 3 shows the effect of the attention mechanism. We trained the model with attention, without attention, and finally without BGRU. We see that attention helps to reduce the error rates when it is applied with a decoder.

Neural networks use randomness in design to ensure that the function approximated for the problem is effectively learned. Randomness is used because it can provide better performance with a machine learning algorithm than the others. Random initialization of the network weights is the most common type of randomness used in neural networks. Randomness can also be used in other areas. Here are some examples:

- Initialization Randomness, such as weights.
- Regularization randomness, such as dropout.
- Randomness in layers, like embedding of words.
- Optimization randomness, such as stochastic optimization.

### 5.3. Experimental Evaluation on HKR dataset

**Figure 6:**
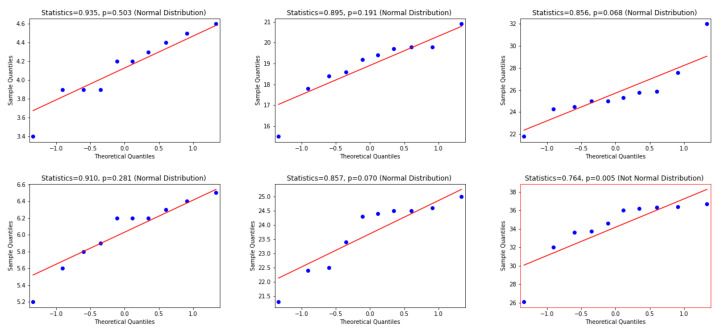
This figure provides Quantile–quantile plots with the *p*-values of the Shapiro–Wilk test for the differences between CER, WER, and SER results in Section 5.2 represents the distributions of Attention-Gated-CNN-BGRU that are computed in terms of test1 and test2 dataset.

**Figure 7:**
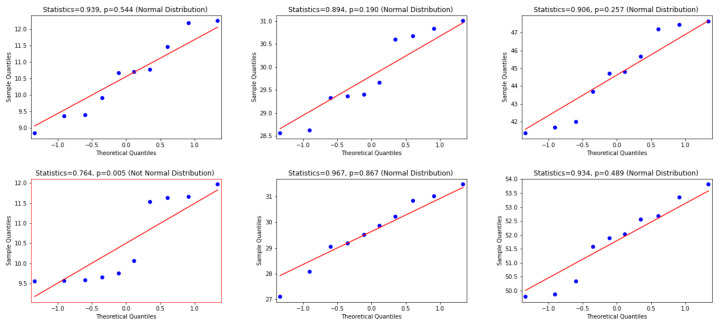
This figure provides Quantile–quantile plots with the *p*-values of the Shapiro–Wilk test for the differences between CER, WER, and SER results in Section 5.2 represents the distributions of Bluche that are computed in terms of test1 and test2 dataset.

**Figure 8:**
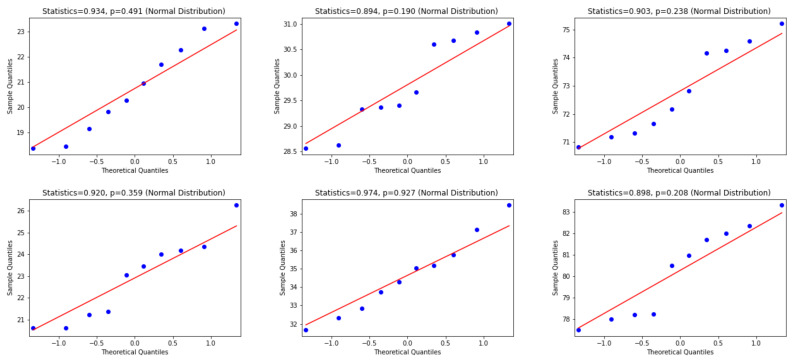
This figure provides Quantile–quantile plots with the *p*-values of the Shapiro–Wilk test for the differences between CER, WER, and SER results in Section 5.2 represents the distributions of Puigcerver that are computed in terms of test1 and test2 dataset.

To use the appropriate statistical test, we examine whether the differences between WER and CER results are normally distributed or not. Consequently, the Quantile—Quantile (Q-Q) plot [48], the Shapiro—Wilk test [49] and D’Agostino and Pearson’s Test [50] are used. In this regard, according to visual inspection in the Q-Q plot, a sample is considered to be consistent with a normal distribution if the sample and theoretical quantiles fall close to the line representing the theoretical distribution. Additionally, this decision is supported by an assessment of whether the points fall inside the envelope of 95% pointwise confidence intervals [51]; thus, we inspect the *p*-value that is yielded by the Shapiro—Wilk test if it has a significant result (*p*-value < 0.05), and hence this shows that the data significantly deviate from a normal distribution. Accordingly, we look for results with a larger *p*-value (i.e., *p*-value > 0.05) to verify that the distribution of the underlying sample in the analysis is normally distributed. Figure 6, Figure 7 and Figure 8 present the distributions of the differences between Attention-Gated-CNN-BGRU and other competitors that are computed based on the two test datasets. According to these figures, the red border image in the figure represents the nonnormal distribution, where one of the points crossed the boundary of the envelope of 95% pointwise confidence intervals and the *p*-value is less than 0.05. All the distributions are normally distributed since the points fall inside the envelope of 95% pointwise confidence intervals and the *p*-value > 0.05. Thus, as the distributions of the underlying differences in the analysis are normally distributed, we favor using the paired t-test with the confidence of 95% (*p*-value< 0.05) to find the statistical significance of the results.

In HKR dataset, we performed ten training executions for each model for statistical testing and used a *t*-test with 5% significance. As null hypothesis we considered $H_{0}$: $\mu_{1}$≥$\mu_{2}$, and an alternative hypothesis $H_{0}$: $\mu_{1}$ < $\mu_{2}$. We analyzed the hypotheses for both the CER, WER, and SER scenarios, where $\mu_{1}$ is the average of the proposed model’s errors, and $\mu_{2}$ is the average of the errors of the other model in comparison. This means that the *p*-value must be lower than α= 0.05 to assume that the proposed model offers a significantly lower error rate.

In both Test 1 and Test 2 datasets, we computed the CER *p*-value and *t* -test, WER *p*-value and *t*-test, and SER *p*-value and *t*-test lower than 0.001. This is below the standard *p*-value = 0.05, meaning that we can conclude that the proposed model, based on Attention-Gated-CNN-BGRU, has a significantly lower CER, WER, and SER in the test1 and test2 dataset. The *p*-values in case of test1 and test2 dataset shown in Table 4.

Another way we used it to prove that our proposed model has a significantly lower CER, WER, and SER. It is called Wilcoxon Signed-Ranks Test [52]. Wilcoxon test is a nonparametric test designed to evaluate the difference between two treatments or conditions where the samples are correlated. It is particularly suitable for evaluating the data from a repeated-measures design in a situation where the prerequisites for a dependent samples t-test are not met. We have calculated both a W-value and a z-value. If N’s size is at least 20, then the distribution of the Wilcoxon W statistic tends to form a normal distribution. This means we can use the z-value to evaluate our hypothesis. If, on the other hand, N’s size is small, and particularly if it is below 10, we should use the W-value to evaluate our hypothesis. The value of z is −2.8031, and the *p*-value is 0.00512. The result is significant at *p* < 0.05.

### 5.4. Comparison with Other Models on HKR Dataset Using Character Accuracy Rates

We evaluated the results of Attention-Gated-CNN-BGRU and the other models using another method called Character Accuracy Rates(CAR) [53,54], this method is implemented to calculate the accuracy of symbols on Test1 and Test2 dataset.

SimpleHTR originally inspired by ANN architectures by [55] and [9], Harald Scheidl proposed a new approach to HWR task [56] in 2018. The model’s architecture consists of 5 CNN layers, 2 RNN (LSTM) layers, CTC loss and decoder layers.

LineHTR model [57] is just an extension of the previous SimpleHTR model, which was developed to enable the model to process images with a full text line (not a single word only), thus, to increase the model’s accuracy further. Architecture of LineHTR model is quite similar to SimpleHTR model, with few differences at the number of CNN and RNN layers and size of those layers’ input: it has 7 CNN and 2 Bidirectinal LSTM (BLSTM) RNN layers.

According to the authors of “Nomeroff Net” automatic number plate recognition system [58], the OCR architecture solution was originally taken from [59]. Although Nomeroff Net OCR architecture was designed to recognize "machine typed" car numbers, it is also worth to check the model’s performance on handwritten text recognition tasks.

**Figure 9:**
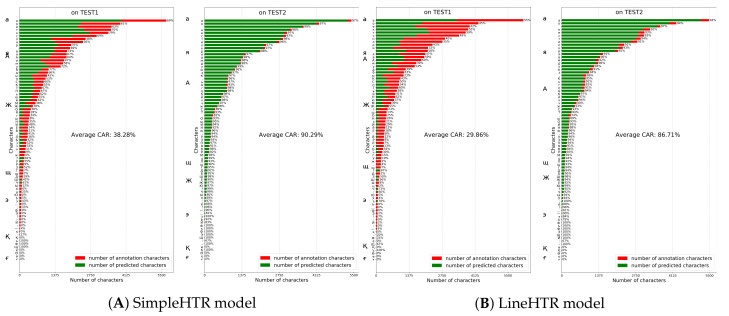
SimpleHTR and LineHTR models performance on TEST1 and TEST2 datasets.

The first experiment was conducted with SimpleHTR model which showed a performance with average Character Accuracy Rates (CAR) of 38.28% and 90.29% on TEST1 and TEST2 datasets respectively (Figure 9). This considerable difference in CAR rates shows that SimpleHTR model overfitted to words seen in training stage and demonstrated lower level of generalization.

Next experiment was carried out with LineHTR model which was trained on data for 100 epochs. This model demonstrated a performance with average CARs of 29.86% and 86.71% on TEST1 and TEST2 datasets respectively (Figure 9). Similar tendency of overfitting to training data can be observed here as well.

**Figure 10:**
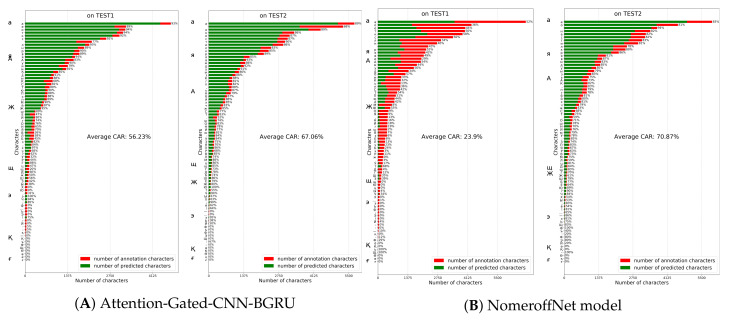
Attention-Gated-CNN-BGRU and NomeroffNet models performance on TEST1 and TEST2 datasets.

**Figure 11:**
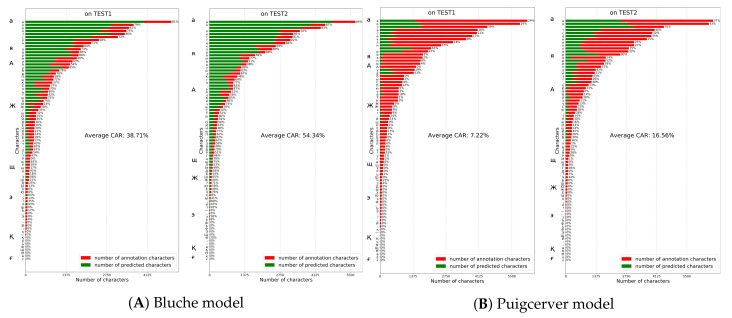
Bluche and Puigcerver models performance on TEST1 and TEST2 datasets.

The same experiments were conducted with NomeroffNet HTR model. Unlike previous models examined, this model showed lower average CAR rate (70.87%) even on TEST2 dataset (Figure 10). The model’s CAR value on TEST1 dataset was reported as 23.9%. As can be observed from the figure, NomeroffNet model also suffers from overfitting.

the last experiment was for Attention-Gated-CNN-BGRU, Bluche and Puigcerver models. Bluche model achieved 38.71% and 54.34% on TEST1 and TEST2 datasets (Figure 11). The PuigcerverHTR model achieved 7.22% and 16.56% on TEST1 and TEST2 datasets(Figure 11). The Attention-Gated-CNN-BGRU CAR rates on TEST1 and TEST2 datasets were reported as 56.23% and 67.06% respectively (Figure 10). As shown in the figure, Attention-based Fully Gated CNN-BGRU model resulted in higher CAR and generalization rates overall.

### 5.5. Comparison with Other Datasets

The results of our research model will be demonstrated and compared with other public datasets such as IAM [60], Saint Gall [61], Bentham [62], and Washington [63]. The IAM Handwriting Database 3.0 includes: 1539 scanned text pages, 5685 isolated and labeled sentences, 657 authors’ contributed handwriting samples, 13,353 isolated and labeled text lines, and 115,320 isolated and labeled words. This dataset includes training, validation, and test splits, where the validation or test splitting cannot involve an author contributing to the training set.

The Saint Gall database contains a handwritten historical manuscript with the following features: the 9th century, the Latin language, a single writer, Carolingian script, and parchment ink. The Saint Gall database contains 60 pages, 1410 text lines, 11,597 words, 4890 word labels, 5436 word spellings, and 49 letters. The Washington database was developed at the Library of Congress from George Washington Papers and has the following characteristics: the eighteenth century, the English language, two authors, longhand script, and paper ink. The Washington database includes 20 pages, 656 lines of text, 4894 instances of the word, 1471 classes of words, and 82 letters.

Bentham’s writings contain many articles written by renowned British philosopher and reformist Jeremy Bentham (1748–1832). This sequence is currently transcribed by an amateur volunteer involved in the award-winning crowd-sourced initiative, Transcribe

Now more than 6000 documents have been transcribed through this public online site. Bentham data set is a subset of documents transcribed using TranScriptorium. This dataset is free and available in two parts for research purposes: the images and the GT. The GT provides information on each image’s layout and transcription on the line stage in page format. Both sections must be downloaded separately. In each section, a comprehensive explanation is given of how the dataset is structured. We obtained state-of-the-art results on IAM, Saint Gall, Bentham, and Washington’s databases presented in Table 5.

### 5.6. Data Augmentation and Batch Normalization

To artificially supplement the training samples and minimize overfitting, we performed sufficient random distortions on the input images. Also, we have used the data augmentation schemes of Perez [70] like affine, flips, scaling, gray-scale erosion, dilation etc. Table 6 describes the implementation details of each strategies. Those strategies are applied randomly while training the model.

Table 7 shows the impact of data augmentation and batch normalization. When adding random distortions to the input images, batch normalization leads to decrease the error rates as the following:

(1) In the Attention-Gated-CNN-BGRU on IAM, the CER on the test set decreases from 5.79% to 3.23%, and WER decreases from 15.85% to 9.21%. In the Bluche model on IAM, the CER on the test set decreases from 6.96% to 4.94%, and WER decreases from 18.89% to 11.21%. Finally, In the Puigcerver model on IAM, the CER on the test set decreases from 8.20% to 5.97%, and WER decreases from 25.0% to 13.73%.

(2) In the Attention-Gated-CNN-BGRU on Saint Gall, the CER on the test set decreases from 7.25% to 4.47%, and WER decreases from 23.0% to 19.21%. In the Bluche model on Saint Gall, the CER on the test set decreases from 8.13% to 7.01%, and WER decreases from 25.78% to 21.87%. Finally, In the Puigcerver model on Saint Gall, the CER on the test set decreases from 12.50% to 9.15%, and WER decreases from 36.9% to 25.37%.

(3) In the Attention-Gated-CNN-BGRU on Washington, the CER on the test set decreases from 8.70% to 5.32%, and WER decreases from 21.50% to 15.21%. In the Bluche model on Washington, the CER on the test set decreases from 12.78% to 9.90%, and WER decreases from 23.15% to 18.95%. Finally, In the Puigcerver model on Washington, the CER on the test set decreases from 20.60% to 14.29%, and WER decreases from 35.6% to 24.87%.

(4) In the Attention-Gated-CNN-BGRU on Bentham, the CER on the test set decreases from 6.10% to 4.73%, and WER decreases from 18.20% to 11.18%. In the Bluche model on Bentham, the CER on the test set decreases from 7.81% to 5.40%, and WER decreases from 20.93% to 15.82%. Finally, in the Puigcerver model on Bentham, the CER on the test set decreases from 7.20% to 5.56%, and WER decreases from 20.3% to 16.75%.

### 5.7. Experimental Evaluation on Other Datasets

In each dataset (IAM, Saint Gall, Washington, and Bentham), we performed ten training executions for each model for statistical testing and we used a *t*-test with 5% significance. As null hypothesis we considered $H_{0}$: $\mu_{1}$≥$\mu_{2}$, and an alternative hypothesis $H_{0}$: $\mu_{1}$ < $\mu_{2}$. We analyzed the hypotheses for both the CER, and WER scenarios, where $\mu_{1}$ is the average of the proposed model’s errors, and $\mu_{2}$ is the average of the errors of the other model in comparison. This means that the *p*-value must be lower than α= 0.05 to assume that the proposed model offers a significantly lower error rate.

We computed the CER *p*-value and *t*-test, and WER *p*-value and *t*-test lower than 0.0001 in test datasets. This is below the standard *p*-value = 0.05, meaning that we can assume that the proposed model, based on Attention-Gated-CNN-BGRU, has a significantly lower CER, WER, and SER in the test datasets. The *p*-values in case of test datasets shown in Table 8.

### 5.8. Discussion

This research’s primary goal was to study and quantitatively compare the state-of-the-art RNN models to choose the best one in the task of handwritten Cyrillic postal- address recognition. This goal also incorporates all efforts put into improving the best performing RNN model. According to the experimental results, Attention-Gated-CNN-BGRU demonstrated comparatively better results in terms of generalization and overall accuracy (see Table 1). As the dataset includes a small number of Kazakh language handwritings, the language characters have lower frequencies (distribution in the dataset) compared to other Cyrillic letters. Consequently, the models mentioned above struggle to recognize these characters, which gives low error recognition rates. Hence, this affects the overall average CAR rates. The dataset also includes non-alphabetic characters (such as “. , !” and so on) with small distributions. Puigcerver models seemed prone to overfitting while being trained to Cyrillic handwriting. We suppose that enrichment of the dataset with various Kazakh and Russian words will solve this problem.

In the proposed architecture, the “image-level” consisting of convolutions and a language-level model of recurrent layers has been conceptually separated. Attention-Gated-CNN-BGRU has been trained for more than ten times, and all results for each experiment of training, evaluation, and testing in the different random seed are recorded in Table 9.

By training the encoder on a large volume of Russian and Kazakh dataset from various collections, we plan in the future to use Attention-Gated-CNN-BGRU for other applications, including speech recognition, image tagging, video captioning, sign language translation, music composition, and genome sequencing, which may benefit from our approach. For example, a recurrent neural network transforms raw voice into character streams using the DeepSpeech speech recognition technique. Both streams of characters use CTC for logical words in text streams.

## 6. Conclusions

In this paper, we have considered the use of encoder–decoder neural network architecture to achieve state-of-the-art results for Kazakh and Russian handwriting recognition. It consists of a fully gated convolutional encoder that extracts generic features of handwritten text, an attention mechanism, a BGRU decoder, and a CTC model to predict the sequence of characters. An important block is the attention mechanism that increases the network’s capacity to collect the essential information for every part of the output sequence and gated layers implemented in the encoder, which can select essential features and inhibit the others.

Our method achieves a high level of recognition rate with a small number of parameters. The experiments results empirically evaluates the performance of the proposed method and it achieves state-of-the-art results on the English-based IAM, Saint Gall, Bentham, Washington, and the Russian–Kazakh dataset(HKR). Future work includes experimentation with larger contextual filters, the addition of sentence-level features like sent2vec, the introduction of hierarchical processing to achieve a high recognition rate at the symbol, word, sentence, and paragraph level, usage of fully convolutional networks, to be able to apply these methods to more languages, resource-constrained languages, and other recurrent cases.

## Figures and Tables

**Table 1:** Comparison of results on HKR dataset to previous methods.

Model	Test1			Test2			Params
	CER (in %)	WER (in %)	SER (in %)	CER (in %)	WER (in %)	SER (in %)	
**Attention-Gated-CNN-BGRU**	**4.13**	**18.91**	**25.72**	**6.31**	**23.69**	**35.16**	885K
	**[3.4–4.6]**	**[15.5–20.9]**	**[21.8–32.0]**	**[5.2–6.4]**	**[21.3–25.0]**	**[32.0–36.7]**	
Bluche [35]	10.55	29.80	44.61	10.50	29.63	51.74	728K
	[8.35–13.1]	[26.9–31.6]	[40.3–48.2]	[9.5–12.2]	[29.45–31.8]	[49.5–55.8]	
Puigcerver [37]	20.73	33.93	72.82	22.91	34.64	80.26	9.6M
	[17.78–23.87]	[31.57–38.84]	[69.56–75.85]	[20.31–26.73]	[31.6–39.2]	[77.2–83.3]	

**Table 2:** Predictions obtained on the HKR dataset.

Input	Label	Prediction
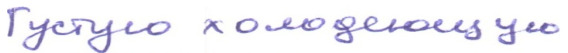	Густую холодеющую	Густую холодеюую
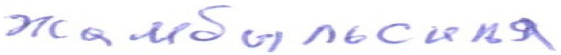	ЖАМБЫЛЬСКАЯ	ЖаМбЫЛСКАЯ
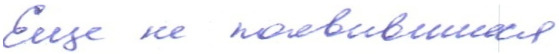	Еще не появившиеся	Еице не появившс
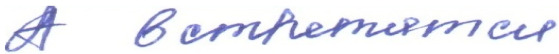	А встретятся	А встретятся
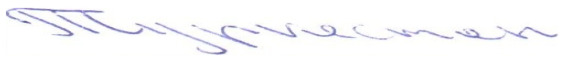	ТУРКЕСТАН	Туркестан
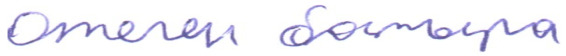	Отеген батыра	Отеген батыра
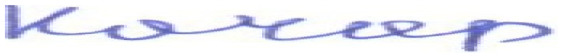	Качар	Качар
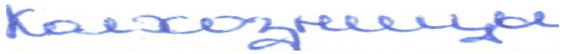	Колхозницы	Колхозницы
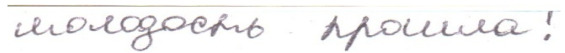	молодость прошла!	молодость прошла !
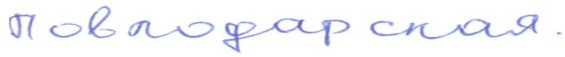	Павлодарская	Павлодарская
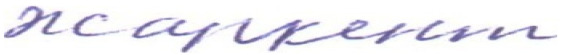	Жаркент	Жаркент
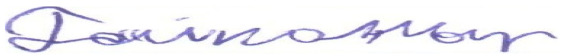	Байконыр	Байконыр

**Table 3:** The effect of attention mechanism on the HKR dataset.

Model	Test1			Test2			Params
	CER (in %)	WER (in %)	SER (in %)	CER (in %)	WER (in %)	SER (in %)	
**Attention model**	**4.13**	**18.91**	**25.27**	**6.31**	**23.69**	**35.16**	**885K**
Without Attention	9.5	37.7	46.1	11.4	41.1	49.4	823K
Without GRU	23.5	76.4	83.6	19.5	61.6	80.8	296K

**Table 4:** A comparison of the recall (i.e., sensitivity) of the CER, WER, and SER for test1 and test2. To verify whether our proposed model achieves statistically significant improvements in the sensitivity of reducing the errors, the paired t-test is conducted with the confidence of 95% (*p*-value < 0.05).

Model		Test1			Test2		
		CER	WER	SER	CER	WER	SER
Attention-Gated-CNN-BGRU—Bluche	*t*-test	−14.36	−20.38	−13.64	−12.29	−9.70	−23.69
	*p*-value	1.63 × 10${}^{-}$${}^{7}$	7.66 × 10${}^{-}$${}^{9}$	2.56 × 10${}^{-}$${}^{7}$	6.23 × 10${}^{-}$${}^{7}$	4.60 × 10${}^{-}$${}^{6}$	2.02 × 10${}^{-}$${}^{9}$
Attention-Gated-CNN-BGRU—Puigcerver	*t*-test	−27.03	−26.85	−43.74	−27.39	−12.51	−70.11
	*p*-value	6.26 × 10${}^{-}$${}^{10}$	6.66 × 10${}^{-}$${}^{1}0$	8.51 × 10${}^{-}$${}^{12}$	5.57 × 10${}^{-}$${}^{10}$	5.397 × 10${}^{-}$${}^{7}$	1.23 × 10${}^{-}$${}^{13}$

**Table 5:** Comparison of results on public datasets.

Model	IAM		Saint Gall		Washington		Bentham	
	CER (in %)	WER (in %)	CER (in %)	WER (in %)	CER (in %)	WER (in %)	CER (in %)	WER (in %)
Graves [64]	18.20	25.90	-	-	-	-	-	-
Almazan [65]	11.27	20.01	-	-	-	-	-	-
Chen [66]	11.15	34.55	-	-	-	-	-	-
Pham [67]	10.80	35.10	-	-	-	-	-	-
Krishnanet [68]	9.78	32.89	-	-	-	-	-	-
Chowdhury [41]	8.10	16.70	-	-	-	-	-	-
Jorge Sueiras [69]	6.20	12.70	-	-	-	-	-	-
Lei Kang [40]	6.88	17.45	-	-	-	-	-	-
Puigcerver [37]	8.20	25.0	12.5	36.9	20.6	35.6	7.2	20.3
Bluche [35]	6.96	18.89	8.13	25.78	12.78	23.15	7.81	20.93
**Attention-Gated-CNN-BGRU**	**5.79**	**15.85**	**7.25**	**23.0**	**8.70**	**21.50**	**6.10**	**18.20**

**Table 6:** Data augmentation strategies.

	Method	Description
1	Saturation, Contrast, and Brightness	To modify saturation, contrast, and brightness, we sampled random factors from a uniform distribution of [0.7, 1.3].
2	Saturation, Contrast, Brightness, and Hue	As shown in 1, but also the hue is shifted by a value sampled from a uniform distribution of [−0.1, 0.1].
3	Affine and Scaling	Rotate the image by up to 90, shear by up to 20, and scale the area by [0:8; 1:2]. New pixels are filled symmetrically at edges.
4	Flips	Randomly flip the images horizontally and/or vertically.
5	Random Crops	Randomly crop the original image. The crop has 0.4–1.0 of the original image, and 3/4–4/3 of the original aspect ratio.
6	Random Erasing	Fill part of the image (area up to 30% of the original image) with random noise. The transformation is applied with a probability of 0.5.
6	Erosion and Dilation	gray-scale erosion with 5%, and dilation with 5%.

**Table 7:** The effect of data augmentation and batch normalization on other datasets.

Model	IAM		Saint Gall		Washington		Bentham	
	CER (in %)	WER (in %)	CER (in %)	WER (in %)	CER (in %)	WER (in %)	CER (in %)	WER (in %)
Puigcerver	5.97 ± 0.06	13.73 ± 0.17	9.15 ± 0.06	25.37 ± 0.15	14.29 ± 0.09	24.87 ± 0.19	5.65 ± 0.07	16.75 ± 0.18
Bluche	4.94 ± 0.07	11.19 ± 0.19	7.01 ± 0.04	21.87 ± 0.15	9.90 ± 0.07	18.95 ± 0.18	5.40 ± 0.06	15.82 ± 0.15
**Attention-Gated-CNN-BGRU**	**3.23** ± **0.03**	**9.21** ± **0.11**	**4.47** ± **0.05**	**19.21** ± **0.12**	**5.32** ± **0.06**	**15.87** ± **0.14**	**4.73** ± **0.04**	**11.18** ± **0.13**

**Table 8:** A comparison of the recall (i.e., sensitivity) of the CER, WER, and SER for test dataset on (IAM, Saint Gall, Washington, and Bentham). To verify whether our proposed model achieves statistically significant improvements in the sensitivity of reducing the errors, the paired *t*-test is conducted with the confidence of 95% (*p*-value < 0.05).

Model		IAM		Saint Gall		Washington		Bentham	
		CER	WER	CER	WER	CER	WER	CER	WER
Attention-Gated-CNN-BGRU—Bluche	*t*-test	−80.26	−44.08	−164.48	−94.58	−465.65	−74.68	−54.84	−129.43
	*p*-value	3.65 × 10${}^{-}$${}^{14}$	7.94 × 10${}^{-}$${}^{12}$	5.76 × 10${}^{-}$${}^{17}$	8.36 × 10${}^{-}$${}^{15}$	4.94 × 10${}^{-}$${}^{21}$	6.99 × 10${}^{-}$${}^{14}$	1.12 × 10${}^{-}$${}^{12}$	4.98 × 10${}^{-}$${}^{16}$
Attention-Gated-CNN-BGRU—Puigcerver	*t*-test	−150.39	−96.58	−819.12	−221.11	−488.43	−257.28	−71.33	−125.74
	*p*-value	1.29 × 10${}^{-}$${}^{16}$	6.93 × 10${}^{-}$${}^{15}$	3.06 × 10${}^{-}$${}^{23}$	4.02 × 10${}^{-}$${}^{18}$	3.21 × 10${}^{-}$${}^{21}$	1.03 × 10${}^{-}$${}^{18}$	1.05 × 10${}^{-}$${}^{13}$	6.46 × 10${}^{-}$${}^{16}$

**Table 9:** CER, WER, SER for Test1, and Test2 for 10-time experiments.

Exp	Test1			Test2			Seed	Time
	CER (in %)	WER (in %)	SER (in %)	CER (in %)	WER (in %)	SER (in %)		
1	4.5	19.2	25.3	6.4	24.5	36.1	1234	3 days, 2:52:28
2	4.6	20.9	27.6	6.3	24.5	36.3	50	2 days, 22:22:27
3	3.9	17.8	24.3	6.5	25.0	36.7	70	4 days, 14:32:49
4	4.2	19.8	25.0	6.2	24.3	36.0	1225	7 days, 1:46:26
5	3.9	19.7	32.0	5.8	22.5	33.7	80	5 days, 0:13:21
6	4.2	19.4	25.8	6.2	24.6	36.4	500	3 days, 12:50:51
7	3.4	15.5	21.8	5.2	21.3	32.0	2000	2 days, 10:50:32
8	4.4	18.6	25.0	5.6	22.4	33.6	1334	2 days, 22:36:42
9	4.3	19.8	25.9	6.2	24.4	36.2	800	4 days, 6:15:17
10	3.9	18.4	24.5	5.9	23.4	34.6	150	5 days, 4:52:44
mean	4.13	18.91	25.72	60.30	23.69	35.16	-	-
